# Œdème vulvaire massif pendant la grossesse: à propos d'un cas

**DOI:** 10.11604/pamj.2014.19.338.5572

**Published:** 2014-11-28

**Authors:** Moulay Elmehdi El Hassani, Farid Kassidi, Youssef Benabdejlil, Jaouad Kouach, Driss Rahali Moussaoui, Mohammed Dehayni

**Affiliations:** 1 Service de Gynécologie-Obstétrique, Polyclinic Elrapha, Libreville, Gabon; 2 Service de Gynécologie-Obstétrique, Hôpital Militaire d'Instruction Mohammed V, Rabat, Maroc

**Keywords:** Œdème vulvaire massif, œdème vulvaire sévère, pré éclampsie, grossesse, postpartum, massive vulval edema, severe vulval edema, preeclampsia, pregnancy, postpartum

## Abstract

L’œdème vulvaire massif est rare pendant la grossesse, mais requiert une attention particulière car il peut se greffer de complications maternelles et fœtales. Il peut être associé à plusieurs pathologies spécifiques ou non spécifiques à la grossesse dont le diagnostic fait appel obligatoirement à un interrogatoire et un examen clinique minutieux, puis à un bilan biologique standard. Le traitement doit être étiologique chaque fois que possible à coté du traitement symptomatique. Cette situation peut nécessiter un accouchement par césarienne. En dehors du risque potentiel de nécrose tissulaire et du risque exceptionnel de décès maternel associé à l’œdème vulvaire massif du post-partum l’évolution est favorable sous traitement bien conduit.

## Introduction

L’œdème vulvaire massif chez la femme enceinte est rare, mais requiert une attention particulière car il peut se greffer de complications maternelles et fœtales. Nous rapportons un cas d’œdème massif vulvaire chez une femme enceinte avec une revue des diagnostics différentiels, des étiologies, des complications potentielles et des options thérapeutiques.

## Patient et observation

**Figure 1:**
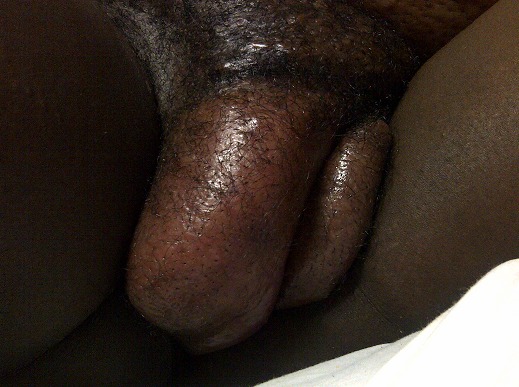
Important œdème vulvaire prédominant à droite

Patiente de 30 ans, deuxième geste, porteuse d'un utérus cicatriciel, sans autres antécédents pathologiques particuliers, admise pour une pré-éclampsie sévère et une menace d'accouchement prématurée sur une grossesse gémellaire évolutive de 32 semaines d'aménorrhée (SA) avec un important œdème vulvaire d’évolution rapide entrainant une fissure de la grande lèvre droite (Figure 1). La patiente était apyrétique, sans notion de traumatisme vulvaire, d'infection ou de prise médicamenteuse. L'examen à l'admission, avait montré une tension artérielle à 170/105 mm Hg, une protéinurie à trois croix à la bandelette urinaire. L'examen de la vulve avait montré un œdème vulvaire massif intéressant les petites et les grandes lèvres, prédominant à droite avec fissuration de la face interne de la grande lèvre droite laissant couler un liquide en eau de roche mouillant les draps. L'examen obstétrical avait montré une hauteur utérine supérieure à l’âge de la grossesse (en rapport avec la grossesse gémellaire), un col effacé à 80% dilaté à 2 cm et une poche des eaux intacte avec un premier jumeau (J1) en siège. L'enregistrement du rythme cardiaque fœtal des deux jumeaux n'avait pas montré d'anomalies. Le reste de l'examen n'avait pas montré de signes de thrombose ni d'adénopathies régionales en dehors d'un œdème des chevilles rétro-maléolaire peu important. Une césarienne a été réalisée pour pré-éclampsie sévère, grossesse gémellaire avec J1 en siège et utérus cicatriciel. Elle a permis l'extraction de deux nouveau-nés de sexe féminin pesant respectivement 1900g et 2000g, Apgar 10/10, confiés au pédiatre. Le bilan biologique à l'admission avait montré une légère anémie hypochrome microcytaire (Hémoglobine à 10,2 g/dl), une thrombopénie à 135000 plaquettes par mm^3^, un taux de prothrombine et des transaminases normaux, un bilan infectieux négatif, une hypoprotidémie à 20g/L et une protéinurie de 24h à 3g/24h. L’œdème vulvaire massif est alors expliqué par l'hypoprotidémie secondaire à l'atteinte rénale dans le cadre de la pré-éclmaplsie sévère. Le traitement avait consisté en des soins locaux au sulfate de magnésium associés à une augmentation de l'apport journalier en protéines en plus de l'arrêt de la grossesse par césarienne qui traite l’étiologie et favoriserait le retour veineux. L’évolution était marquée par la normalisation rapide des chiffres tentionnels et une régression spectaculaire de l’œdème vulvaire, et sa disparition complète en dix jours. Les nouveaux nés ont quitté le service de néonatologie à J7 de vie.

## Discussion

Un œdème peut être observé chez 80% des femmes enceintes [1], mais l’œdème vulvaire massif isolé est rare pendant la grossesse [2], son association à une pré-éclampsie n'a été rapportée dans la littérature que dans quelques cas. L’œdème vulvaire pendant la grossesse peut être associé à plusieurs pathologies notamment: une pré-éclampsie, des grossesses multiples, un traitement tocolytique, un diabète, une dystocie, une deuxième phase du travail prolongée, l'utilisation de chaise d′accouchement comme il peut s'agir d'un œdème vulvaire idiopathique du post-partum. Il peut aussi se rencontré dans d'autres situations non spécifiques à la grossesse [2–4]. L’œdème vulvaire massif du post-partum immédiat requiert une attention particulière car il peut se compliquer d'un collapsus cardiovasculaire et d'un décès maternel [5]. Une tumeur maligne ou bénigne, collection hématique, kyste de Bartholin, hernie inguinale ou d'autres pathologies rares (adénomyose du ligament rond, sein surnuméraire) peuvent simuler un œdème vulvaire [2–4]. L'apparition d’œdèmes au cours d'une grossesse normale est multifactorielle et implique l'activation du système rénine-angiotensine, l’œstrogène et la compression de la veine cave inférieure par le volume utérin. Dans la pré-éclampsie l'augmentation de la pression capillaire et la diminution de la pression oncotique par hypo-albuminémie ramène l'eau dans le milieu interstitiel [1, 3]. La formation de l’œdème massif de la vulve serait dû à sa déclivité en position couchée et à sa richesse en tissu conjonctif lâche avec une mince couche épithéliale [2, 3]. L’œdème vulvaire massif chez notre patiente est probablement dû à l'hypo-protidémie souvent associée à la pré-éclampsie sévère. La grossesse gémellaire étant encore à 32 SA et l'anémie n’étant pas profonde. Le traitement vise le soulagement de la douleur et l'inconfort et l’éviction des complications locales. Il doit être étiologique chaque fois qu'une cause sous-jacente est retrouvée. Pour le mode d'accouchement la césarienne peut être nécessaire si l'accouchement est urgent [3]. En dehors du risque potentiel de nécrose tissulaire [3], et des quelques cas exceptionnels de décès maternel associé à l’œdème vulvaire massif du post-partum [2], l’évolution est favorable sous traitement bien conduit.

## Conclusion

L’œdème vulvaire massif est rare pendant la grossesse mais requiert une attention particulière car il peut se greffer de complications maternelles et fœtales. Le traitement est symptomatique et étiologique chaque fois qu'une cause sous-jacente est retrouvée et l’évolution est souvent favorable sous traitement bien conduit. Un accouchement par césarienne peut être nécessaire.
